# Single mutidrug resistant enterobacteriacae donor-derived infection in four solid organ transplant recipients: a case report

**DOI:** 10.1186/s12893-019-0574-9

**Published:** 2019-08-14

**Authors:** Eva Kieslichova, Marek Protus, Dana Nemcova, Eva Uchytilova

**Affiliations:** 1 Department of Anesthesiology and Intensive Care, Transplantcentre, Institute for Clinical and Experimental Medicine, Prague, Czech Republic; 2 Laboratory Methods Division - Department of Clinical Microbiology, Institute for Clinical and Experimental Medicine, Prague, Czech Republic

**Keywords:** Donor-derived infection, Multi-drug resistant bacteria, Early posttransplant infection

## Abstract

**Background:**

Bacteraemia of the donor is not considered to be contraindication of organ procurement. On the other hand, infection of solid organ transplant recipients remains to be a major cause of their morbidity and mortality. When using organs from bacteraemic donors, individual risks need to be assessed and the appropriate antibiotic treatment applied.

**Case presentation:**

In this case series we report several serious donor–derived infectious complications in four out of five recipients of different organs from one single donor in the early posttransplant period. Donor-transmitted multi-drug resistant strains of *Escherichia coli* and *Klebsiella pneumonia* was confirmed by both serologic and molecular testing.

**Conclusions:**

To prevent donor-derived infections, careful microbiological screening followed by targeted antibiotic treatment is essential. Although such complications can never by completely prevented, a high index for potential bacterial infection in organ donors and transplant recipients should be routinely employed.

## Background

Solid organ transplantation (SOT) is the only way to treat end-stage organ disease [1]. However, as the number of potential recipients continues to exceed the number of donated organs from different sources, an imbalance between the number of available organs and potential recipients remains. This increase in demand has led to the extension of donor or organ criteria and a rise in the use of organs from “marginal donors” of older age and those with comorbidities, including infections. Individual risk assessment is therefore essential for ensuring successful outcomes [2].

Even when potential organ donors are not initially infected upon admission to an intensive care unit (ICU), the risk of infection from ensuing invasive procedures can increase significantly. This risk of infection and subsequent organ contamination must be considered before procurement [3]. However, bacteraemia or bacterial sepsis is not considered an absolute contraindication to organ donation provided adequate antibiotic treatment is administered [4, 5].

On the other hand, infection and sepsis are among the most common causes of morbidity and mortality in patients after SOT. Risk of infection is not only based on immunosuppression, epidemiologic exposure and invasive procedures performed in the recipient [6, 7], but also on possible pathogen transmission from the donor.

In this case series, we report donor-derived infection caused by multidrug-resistant (MDR) bacteria *Escherichia coli* producing extended spectrum β-lactamase (ESBL) and *Klebsiella pneumonia* ESBL in four SOT recipients transplanted in one transplant centre (TC).

### Case presentation

In November 2014, a 53-year-old woman with a history of depression was admitted to a regional hospital after resuscitation as a result of a suicide attempt by strangulation, where hypoxia led to the development of a malignant brain oedema. She was afebrile on admission and showed a normal chest radiograph along with microbially negative urine and tracheal secretion. On the sixth day of hospitalisation, she showed clinical signs of bacterial infection (CRP 93.4 mg/l, leukocyte count 17.3 × 10^9^/l), with a chest X-ray showing suspected infiltrate in the basal part of the right lung. A standard microbiological examination was then carried out in conjunction with empiric antibiotic therapy (amoxicillin clavulanate 1.2 g á 8 h i.v.). Forty-eight hours later, *Klebsiella pneumoniae* and *Escherichia coli*, susceptible to all tested antibiotics except ampicilin - i.e. strains which did not meet generally accepted multidrug-resistance criteria, without ESBL production - were detected in the sputum along with microbially negative urine. Not having any pre-existing diseases, after 10 days in the ICU on mechanical ventilation, she was identified as a potential brain-dead organ donor and transported to the TC for further examination (echocardiography, coronarography), instrumental confirmation of brain death and possible organ procurement.

On admission to the TC, the patient was mechanically ventilated, achieving adequate ventilation and oxygenation status (tidal volume 500 ml, respiratory rate 12/min, FiO2 0.4, SpO2 99%); perfusion pressure was maintained with norepinephrine at a maximum dose of 0.05 mcg/kg/min. Laboratory results indicated an increase in inflammatory parameters except for procalcitonin (CRP 227 mg/l, leukocyte count 17.3 × 10^9^/l, PCT 0.35 ng/ml), requiring blood cultures to be sent for microbiological examination before organ procurement, no other samples were taken. However, since she was afebrile and her chest radiograph was normal, there was no clinical evidence of infection. Because organ procurement was initiated just 4 h after admission and the results of the new microbiological examination were not available at that stage, no changes were applied to the primary antimicrobial therapy given to the donor. ICU care of the donor and multi-organ procurement were performed in the standard manner according to current guidelines, with the heart, liver, kidneys and pancreas all procured for islet isolation.

The organs were transplanted to 5 different recipients. All transplantation procedures were performed in the same TC and in a routine manner using standard techniques. Antibiotic prophylaxis (3 doses of cefuroxime 1.5 g i.v. á 8 h for kidney transplantation, piperacillin-tazobactam 4.5 g á 8 h i.v. for 3 days for liver transplantation, piperacillin-tazobactam 4.5 g i.v. single dose before Langerhans islet transplantation, ceftriaxone 1 g á 12 h for 2 days for heart transplantation) and immunosuppressive therapy were applied for all recipients according to routinely used organ-specific protocols and appropriately modified based on the level of immunological risk in each individual patient.

The first kidney recipient was a 60-year-old male on haemodialysis with end-stage kidney disease (ESKD) caused by diabetic nephropathy and a history of tricuspid valve replacement and atrial fibrillation. Since it was his second kidney transplantation, his immunological risk was considered high. The immunosuppression protocol consisted of thymoglobulin, tacrolimus, mycophenolate mofetil and prednisone, with induction by thymoglobulin and methylprednisolone. Cefuroxime 1.5 g á 8 h i.v. was used for antibiotic prophylaxis. On the first postoperative day, the patient suddenly developed haemodynamic instability with signs of both left heart failure and severe sepsis. Immunosuppression therapy was withdrawn and antibiotics were changed to meropenem 2 g á 8 h i.v. and amikacin 1.5 g á 24 h i.v.. Septic shock treatment – inotropes, vasopressors, fluid management and renal replacement therapy – was initiated immediately. On the second postoperative day, surgical intervention was performed due to visceral ischaemia. The patient died on the second day after kidney transplantation secondary to severe septic shock and multi-organ failure (MOF). On the third day after transplantation, microbiology (surgical samples cultures and blood cultures) confirmed the presence of ESBL producing *Klebsiella pneumonia* and ESBL producing *Escherichia coli* in the kidney graft.

The second kidney recipient was a 32-year-old male on peritoneal dialysis with ESKD due to glomerulonephritis and a history of recurrent peritonitis requiring antibiotic treatment. Because this was his third kidney transplantation and his immunological risk was extremely high, plasmapheresis was performed prior to surgery. Immunosuppression was induced by thymoglobulin (3 doses in total according to protocol) and intravenous immunoglobulin, and maintained by tacrolimus, mycophenolate mofetil and prednisone. Cefuroxime 1.5 g á 8 h was initially used as prophylaxis, as in the first kidney recipient. On the first postoperative day, the patient showed signs of bleeding, and on the second postoperative day he suddenly developed both haemorrhagic and septic shock caused by a strangulated sigma loop via the peritoneal catheter. As with the first recipient, immunosuppression therapy was discontinued, meropenem 2 g á 8 h i.v. and metronidazole 500 mg á 8 h i.v. administered, and emergency surgery performed. Despite sepsis treatment, volume substitution including blood products, and renal replacement therapy, the patient died on the third day after transplantation secondary to MOF caused by hypovolemic and septic shock. Microbiological examination (surgical samples cultures, drain secretion cultures) showed the same results as those of the first kidney recipient.

The liver recipient was a 49-year-old male with hepatitis B virus-related cirrhosis with portal hypertension and a Model for End-Stage Liver Disease score of 19. He was admitted to hospital 10 days before transplantation because of a ruptured umbilical hernia resulting in peritonitis, which was treated by cefotaxime for 10 days. Immunosuppression consisted of methylprednisolone induction, tacrolimus, mycophenolate mofetil and prednisone. Antibiotic prophylaxis consisted of piperacillin-tazobactam 4.5 g á 8 h i.v. administered over 3 days. The patient exhibited no postoperative complications. On the second day after transplantation, ESBL-producing *Klebsiella pneumoniae* was found in the drain secretion. Antibiotics were therefore changed to meropenem 1 g á 8 h i.v. for 8 days. At the time of writing, the patient is still alive with good graft function.

Langerhans islets were transplanted to a 59-year-old female with type I diabetes mellitus complicated by daily episodes of hypoglycaemia and chronic kidney disease. Immunosuppression was induced by thymoglobulin and methylprednisolone and maintained by tacrolimus and rapamycin. The recipient was administered piperacillin-tazobactam 4.5 g i.v. single dose for prophylaxis. On the first day after transplantation, she developed severe haemorrhagic shock with extreme blood loss. This was followed by the development of sepsis due to pneumonia on the second day after transplantation, accompanied by acute kidney injury leading to MOF. The patient underwent several emergency surgeries. ICU treatment was based on immunosuppression withdrawal, antibiotic therapy (piperacilin-tazobactam 2.25 g á 6 h i.v. because of extracorporal renal replacement therapy and metronidazole 1 g á 8 h i.v., changed on the 4th day to meropenem 1 g á 6 h i.v. for 10 days and amikacin - dose adjusted according to serum level á 24 h i.v. for 10 days) and treatment of hypovolemic and septic shock, consisting of blood products, mechanical ventilation and haemodialysis. On the second day after transplantation, ESBL-producing *Escherichia coli* was detected in her sputum, and on the third day ESBL-producing *Klebsiella pneumoniae* was found in her blood cultures. After 81 days in-hospital, the patient was discharged despite the failure in function of the transplanted Langerhans islets. She subsequently underwent another, successful islet transplantation.

The heart allograft recipient was a 40-year-old male suffering from dilated cardiomyopathy and cardiorenal syndrome. Immunosuppression consisted of thymoglobulin and methylprednisolone as induction, with thymoglobulin, tacrolimus, mycophenolate mofetil and methylprednisolone for maintenance. Ceftriaxone 1 g á 12 h i.v. for 2 days was initially used for prophylaxis, but because of infectious complications to the other recipients, meropenem 1 g á 6 h was administered on the third post-transplant day, this treatment was maintained for 10 days. No complications or positive microbial findings were recorded for this patient. He is presently alive with good graft function (Table 1).

**Table 1:** Characteristics of individual recipients and their perioperative period

number of patient	age/sex/BMI	age/sex/BMI transplanted organ	principal disease	prior to SOT hospitalization/ATB therapy	immunological risk	immunosuppression	antibiotic prophylaxis	complication	therapy	antibiotic therapy	LOS [days]	outcome
1	60/male/26.6	kidney	chronic kidney disease, diabetic nephropathy, kidney transplantation 4 years ago, early graftectomia due to renal vein thrombosis, cardiac anamnesis: tricuspid valve repair due to regurgitation, atrial fibrillation (warfarine), EF LV 50%), habitual hypotension (midodrine), hemodialysis (AV shunt)	no/no	high 2nd kidney transplantation, PRA act 0%, max 14%	thymoglobuline and methylprednisolone (induction), thymoglobuline, tacrolimus, mycophenolate mofetil, prednisone, withdrawal on the 3rd day	cefuroxime (2 days)	early hemodynamic instability, left heart insuficiency, arythmia, sepsis, visceral ischemia	surgery, inotropes, antiarythmics, therapy of the septic shock acording to guidleines, CVVHD, mechanical ventilation for 48 h	since 2nd postoperative day meropenem 2 g á 8 h, amikacin 2 g á 24 h	3	death on the 2nd day after SOT, sepsis, multiorgan failure
2	32/male/25.1	kidney	chronic kidney disease, chronic glomerulonephritis, previous 2 kidney transplantations, peritoneal dialysis, 2 episodes of peritonitis	no/no	high high level of DSA, PRA act 36%, max 79% plasmapheresis before the transplantation	thymoglobuline (3 doses), intravenous immunoglobuline (3 doses), tacrolimus, mycophenolate mofetil, prednisone	cefuroxime (2 days)	haemorrhage, strangulated sigma loop around CAPD catether, haemorrhagic shock	surgery, therapy of circulatory shock, CVVHD, mechanical ventilation for 52 h	since 2nd postoperative day meropenem 2 g á 8 h, metronidazole 500 mg á 8 h	4	death on the 3th day after SOT, sepsis, multiorgan failure
3	49/male/23.1	liver	HCV related liver cirrhosis Child-Pugh C, portal hypertension, hepatorenal syndrome, ascites, encephalopathy	yes/yes	low, PRA 0%	methylprednisolone (induction), tacrolimus, mycophenolate mofetil, prednisone	piperacilin/tazobactam (3 days)	none	usual postoperative care, early extubation	since 3rd postoperative day meropenem 1 g á 8 h (for 10 days)	22	alive (2018) with good graft function
4	59/female/25.8	pancreas islets	labile diabetes mellitus type I, daily episodes of hypoglycemia, chronic kidney disease, cholestasis of the liver	no/no	low, PRA act 0%, max 13%	thymoglobuline and methylprednisolone (induction), tacrolimus, rapamycine – with drawal on the 2nd day	piperacilin/tazobactam (3 days)	haemorrhage, pneumonia, haemorrhagic and septic shock, acute kidney injury and multiorgan failure	several surgeries, therapy of circulatory shock, CVVHD, intermitent dialysis, mechanical ventilation for 540 h, tracheostomy	piperacilin/tazobactam 2,25 g á 6 h, on the 4th postoperative day changed to meropenem 1 g á 6 h, amikacin acording to serum level (for 10 days)	81	alive (2018), early failure of the transplanted islets, 2nd successfull islets transplantation in 2018
5	40/male/28.4	heart	dilatation cardiomyopathy, EF LV < 20%, pulmonary hypertension, ICD, chronic kidney disease	yes/yes	low, PRA 0%	thymoglobuline, methylprednisolone, and mycophenolate mofetil (induction), thymoglobuline, tacrolimus, methylpredisolone, mycophenolate mofetil	cefriaxone (3 days)	none	usual postoperative care, mechanical ventilation for 13 h	since 2nd postoperative day meropenem 1 g á 6 h (for 10 days)	40	alive (2018), good graft function

**Fig. 1:**
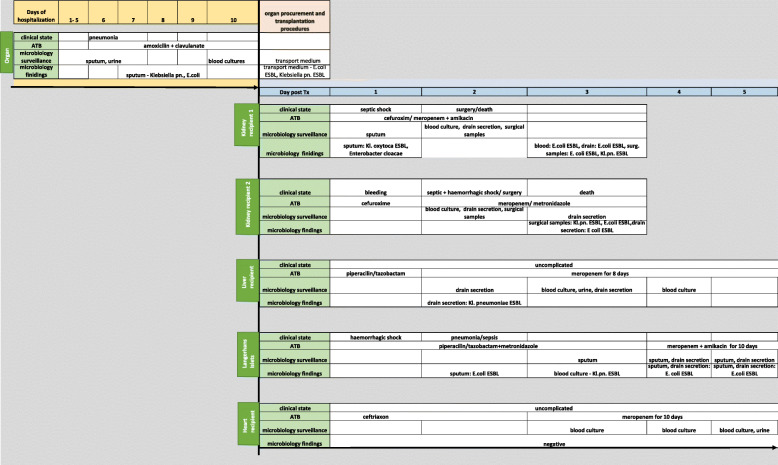
Timeline of case presentation

In summary, 4 out of 5 recipients developed donor-derived infectious complications within 48 h post-transplant (Fig. 1). While bacteria of good susceptibility were found during the primary microbiological examination of the donor on the sixth day in ICU, MDR strains (both ESBL *Klebsiella pneumoniae* and ESBL *E. coli*) were found in the transport medium in which the organs (kidney and Langerhans islets with duodenum) were stored during organ procurement, but not in the blood culture taken before procurement. In the samples of both kidney allograft recipients, the Langerhans islet recipient, and the liver recipient, the same MDR bacteria were detected on the third postoperative day. Pulsed field gel electrophoresis confirmed the identity of these bacterial strains. Using PCR the presence of extraintestinal virulence factors pap C, sfa/focCD (adhesins) and gimB (invasin) was detected in ESBL *E. coli*. The graft perfusion solution (Custodiol) was microbially negative. Secondary contamination during organ procurement/transplantation was ruled out (Tables 2 and 3).

**Table 2:** Microbiology examination of the donor’s sputum, including drug sensitivity. *S* sensitive, *R* resistant

ATB	*Klebsiella pneumoniae*	*E.coli*
Ampicilin	R	R
Aminopenicilin/inhibitor	S	S
Piperacilin/tazobactam	S	S
Gentamicin	S	S
Ofloxacin	S	S
Cefotaxime	S	S
Ceftazidime	S	S

**Table 3:** Strains isolated from biological materials of the recipients. *R* resistant, *S* sensitive, *I* indifferent

ATB	*Klebsiella pneumoniae*	*E.coli*
Ampicilin	R	R
Aminopenicilin/inhibitor	R	R
Piperacilin/tazobactam	R	R
Gentamicin	R	R
Amikacin	S	I
Ciprofloxacin	R	I
Cefotaxime	R	R
Ceftazidime	R	R
Meropenem	S	S
ESBL	Positive	Positive

## Discussion and conclusions

While findings of positive blood cultures are quite common among potential organ donors, the transmission rate to recipients is relatively low [8]. Organs from bacteraemic donors result in very few transmitted infections in cases where donors have received pathogen-targeted antibiotics before procurement [9]. According to one study, even when blood culture positivity was not detected in donors with bacteraemia before procurement, neither graft nor recipient survival outcomes differed from those of non-contaminated organ recipients when using pathogen-specific antibiotic treatment [10]. Therefore, given the potential degree of medical urgency on the part of the recipient and the adaptability of antimicrobial prophylaxis based on current microbiological findings, donor infection is not considered a contraindication of organ procurement [11, 12].

However, in the initial stage preceding seroconversion, donor infection often goes undetected due to the limited sensitivity of routine laboratory tests toward pathogen detection [4, 12]. Moreover, most brain-dead organ donors with bacterial infection are afebrile, and thus many exhibit unrecognised or unidentified bacterial infection at the time of donation, increasing the risk to recipients of pathogen transmission [13]. However, although such donor-derived infections are rare, with incidence at approximately 0.2–1.7% of all SOTs, they are often serious and accompanied by significant morbidity and mortality [14–16]. The routine collection of biological material for microbiological examination during organ procurement (sputum, urine, and haemocultures) could be useful in spite of the time pressures on obtaining results.

In the case described above, the urinary tract of the organ donor was colonised by MDR strains of ESBL *Escherichia coli* and ESBL *Klebsiella pneumoniae* at the time of organ procurement, as their drug susceptibility changed after empiric antibiotic therapy, which was confirmed by pulsed field gel electrophoresis. Unfortunately, these strains were not detected in the sputum or in the urine of the donor on the sixth ICU day, while the increase of inflammatory markers at the time of transport may have been attributed to the onset of new infection.

In organ donors, only routine screening of common bacterial and viral infections is performed, while screening of colonization by MDR strains is not. Extended microbiological examination is indicated only if there is reasonable suspicion of infection. Standard cultivation is time – consuming (18–24 h minimally) and the results of fast methods based on polymerase chain reaction are quickly accessible, but expensive with limited specificity, without information about drug susceptibility. In our case, the donor, showing clear signs of infection, had many risk factors for colonization and infection by nosocomial MDR strains. Administration of broad-spectrum antibiotic treatment should have been considered, even though there was no evidence.

The recipients differed with regard to antibiotic prophylaxis, immunosuppression levels and incidence of postoperative complications. Severe bleeding occurred in three cases, most likely associated with the presence of Gram-negative bacteria in the blood stream [17]. All of these factors seem to relate to the possible development of sepsis. Both of the kidney recipients who were placed on the intensive immunosuppression protocol and underwent early surgical revision died because of fulminant septic shock, while the liver, heart and Langerhans islet recipients survived.

We consider this case to be instructive for several reasons. First, it illustrates that the length of time spent in an ICU increases the risk of colonisation by MDR nosocomial strains [18–20], however, there are no specific predictors of ESBL – producing microorganisms colonization [21]. Second, microbiological screening of the donor, despite the absence of clear signs of acute infection, is very important. Third, even though absolute prevention of donor-derived infection is not possible, the risk of transfer can be significantly reduced [4, 5, 12].

Communication between donor centres and their labs, organ procurement organizations and transplant centres is a cornerstone of transplantation medicine. The quality of communication was investigated by Miller and colleagues [22]. Number of infectious events, of which many lead to serious adverse events, including deaths, was registered. Most of these infectious adverse events were caused by communication gaps, as the test results were reported with delay, so appropriate interventions could not be taken.

To conclude, donor screening for the presence of both common and uncommon pathogens should always be carefully performed. Furthermore, any subsequent antibiotic treatment should be adjusted based on results and the local epidemiologic situation, with a focus on MDR bacteria. Good communication among healthcare facilities charged with caring for the donor and recipients can play an important role in expediting the use of targeted antibiotic treatment in infected recipients.

## Data Availability

The datasets used and analysed are available from the corresponding author on reasonable. request.

## Abbreviations

ESBL: extended spectrum β-lactamase
ESKD: end-stage kidney disease
ICU: intensive care unit
MDR: multidrug-resistant
MOF: multi-organ failure
SOT: solid organ transplantation
TC: transplantcentre

## References

[1] Hernández D, Muriel A, Abraira V (2016). Current state of clinical end-points assessment in transplant: key points. Transplant Rev.

[2] McKeown DW, Bonser RS, Kellum JA (2012). Management of the heartbeating brain-dead organ donor. Br J Anaesth.

[3] Kotloff RM, Blosser S, Fulda GJ, Malinoski D, Ahya VN, Angel L (2015). Management of the Potential Organ Donor in the ICU: Society of Critical Care Medicine/American College of Chest Physicians/Association of Organ Procurfement Organizations Consensus Statement. Crit Care Med.

[4] Grossi PA, Fishman JA (2009). AST infectious disease Community of Practice. Donor-derived infections in solid organ transplant recipients. Am J Transplant.

[5] Anesi JA, Blumberg EA, Abbo LM (2018). Perioperative antibiotic prophylaxis to prevent surgical site infections in solid organ transplantation. Transplantation.

[6] Fishman JA, and the AST Infectious Diseases Community of Practice (2009). Introduction. Infection in solid organ transplant recipients. Am J Transpl.

[7] Linden PK (2009). Approach to the immunocompromised host with infection in the intensive care unit. Infect Dis N AM.

[8] Freeman RB, Giatras I, Falagas ME, Supran S, O'Connor K, Bradley J (1999). Outcome of transplantation of organs procured from bacteremic donors. Transplantaiton.

[9] Cohen J, Michowiz R, Ashkenazi T, Pitlik S, Singer P (2006). Successful organ transplantation from donors with Acinetobacter baumannii septic shock. Transplantation.

[10] Lumbreras C, Sanz F, González A, Pérez G, Ramos MJ, Aguado JM (2001). Clinical significance of donor-unrecognized bacteremia in the outcome of solid-organ transplant recipients. Clin Infect Dis.

[11] Fishman JA (2007). Infection in solid-organ transplant recipients. N Engl J Med.

[12] Grossi PA (2018). Donor-derived infections, lessons learnt from the past, and what is the future going to bring us. Curr Opin Organ Transplant.

[13] Fishman JA, Greenwald MA, Grossi PA (2012). Transmission of infection with human allografts: essential considerations in donor screening. Clin Infect Dis.

[14] Ison Michael G. (2009). The Epidemiology and Prevention of Donor-Derived Infections. Advances in Chronic Kidney Disease.

[15] Ison MG, Grossi P (2013). Donor-derived infections in solid organ transplantation. Am J Transplant.

[16] van Duin D, Van Delden C (2013). Multidrug-resistant gram-negative bacteria infections in solid organ transplantation. Am J Transplant.

[17] Orlando G, Di Cocco P, Gravante G, D’Angelo M, Famulari A, Pisani F (2009). Fatal hemorrhage in two renal graft recipients with multi-drug resistant *Pseudomonas aeruginosa* infection. Transpl Infect Dis.

[18] Goldberg E, Bishara J, Lev S, Singer P, Cohen J (2012). Organ transplantation from a donor colonized with a multidrug-resistant organism: a case report. Transpl Infect Dis.

[19] Ariza-Heredia EJ, Patel R, Blumberg EA, Walker RC, Lewis R, Evans J (2012). Outcomes of transplantation using organs from a donor infected with Klebsiella pneumoniae carbapenemase (KPC)-producing K. pneumoniae. Transpl Infect Dis.

[20] Lewis JD, Sifri CD (2016). Multidrug-resistant bacterial donor-derived infections in solid organ transplantation. Curr Infect Dis Rep.

[21] Escolá-Vergé L, Los-Arcos I, Gonzáles-López JJ, Lung M, Bilbao I, Farré M, Pont T (2017). Successful liver transplantation despite donor-transmitted ESBL-producing *Klebsiella pneumoniae* infection: Case report and review of the literature. Transpl Infect Dis.

[22] Miller R, Covington S, Taranto S, Carrico R, Ehsan A, Friedman B (2015). Communication gaps associated with donor-derived infections. Am J Transplant.

